# Serological responses in humans to the smallpox vaccine LC16m8

**DOI:** 10.1099/vir.0.034207-0

**Published:** 2011-10

**Authors:** Benjamin F. Johnson, Yasuhiro Kanatani, Tatsuya Fujii, Tomoya Saito, Hiroyuki Yokote, Geoffrey L. Smith

**Affiliations:** 1 Section of Virology, Faculty of Medicine, Imperial College London, St Mary’s Campus, Norfolk Place, London W2 1PG, UK; 2 National Institute of Public Health, Department of Policy Sciences, 2-3-6 Minami, Wako-shi, Saitama 351-0197, Japan; 3 Self-Defense Force, Central Hospital, Health Department, Management Division, 1-2-24 Ikejiri, Setagaya, Tokyo 154-8532, Japan; 4 Bio-preparedness Research Laboratory, Department of Tropical Medicine and Parasitology, School of Medicine, Keio University, 35 Shinanomachi, Shinjuku, Tokyo 160-8582, Japan; 5 The Chemo-Sero-Therapeutic Research Institute (KAKETSUKEN), 1-6-1 Okubo, Kumamoto 860-8568, Japan

## Abstract

In response to potential bioterrorism with smallpox, members of the Japanese Self-Defense Forces were vaccinated with vaccinia virus (VACV) strain LC16m8, an attenuated smallpox vaccine derived from VACV strain Lister. The serological response induced by LC16m8 to four virion-surface proteins and the intracellular mature virus (IMV) and extracellular enveloped virus (EEV) was investigated. LC16m8 induced antibody response against the IMV protein A27 and the EEV protein A56. LC16m8 also induced IMV-neutralizing antibodies, but unlike the VACV strain Lister, did not induce either EEV-neutralizing antibody or antibody to EEV protein B5, except after revaccination. Given that B5 is the only target for EEV-neutralizing antibody and that neutralization of both IMV and EEV give optimal protection against orthopoxvirus challenge, these data suggest that immunity induced by LC16m8 might be less potent than that deriving from strain Lister. This potential disadvantage should be balanced against the advantage of the greater safety of LC16m8.

Smallpox was eradicated in 1979 by widespread vaccination with vaccinia virus (VACV) and thereafter smallpox vaccination was discontinued (Fenner *et al.*, 1988). However, due to the potential threat of bioterrorism, limited smallpox vaccination programmes have been restarted, and the World Health Organization (WHO) and several nation states are replenishing their smallpox vaccine stockpiles.

The WHO reference smallpox vaccine was the strain Lister, but several other VACV strains were also used including New York City Board of Health (NYCBH/Dryvax), EM-63 and Tian Tan (Fenner *et al.*, 1988). Although these strains protected against smallpox, they could also cause adverse reactions and eczema, immunodeficiency and pregnancy were recognized as contraindications for smallpox vaccination (Lane *et al.*, 1969). The concern about vaccine safety led to the development of attenuated vaccines by empirical passage, such as LC16m8 (Hashizume *et al.*, 1985) or modified vaccinia virus Ankara (MVA) (Stickl & Hochstein-Mintzel, 1971), or by genetic engineering, such as NYVAC (Tartaglia *et al.*, 1992). However, these strains were not used in countries where smallpox was endemic and, consequently, evidence that they protect against smallpox is lacking. Nonetheless, in animal models, they can protect against disease caused by other orthopoxviruses (OPVs), such as monkeypox virus (Earl *et al.*, 2004; Saijo *et al.*, 2006). Here, we have examined the immunogenicity of LC16m8 by evaluating the neutralizing antibody response to both intracellular and extracellular virions and four individual proteins on the surface of these virions. These results were compared with the immunogenicity of the VACV strain Lister.

LC16m8 is a small plaque variant of the VACV strain Lister (Elstree) obtained by repeated passage of Lister in primary rabbit kidney cells at low temperature (Hashizume *et al.*, 1985). LC16m8 is attenuated in animal models and in man (Hashizume *et al.*, 1985; Saito *et al.*, 2009) and was used to vaccinate over 50 000 children in Japan in the 1970s and members of the Japanese Self-Defense Forces between 2002 and 2005 (Kenner *et al.*, 2006; Saito *et al.*, 2009). The small plaque phenotype of LC16m8 is due to a mutation of the *B5R* gene, resulting in the truncation of the ORF after codon 91 (Takahashi-Nishimaki *et al.*, 1991) and expression of a truncated B5 protein (Meseda *et al.*, 2009). This mutation and additional alterations elsewhere in the genome contributed to the attenuated phenotype of LC16m8 (Takahashi-Nishimaki *et al.*, 1991). Spontaneous mutations in the LC16m8 *B5R* gene that restore the plaque size to normal and increase virulence can occur, but this could be prevented by deletion of the entire gene (Kidokoro *et al.*, 2005). Recently, it was demonstrated that whereas T-cells are needed to prevent development of progressive vaccinia in macaques immunized with ACAM200 (a plaque purified derivative of Dryvax), LC16m8 was unable to spread and cause disease even in the absence of T-cells, demonstrating its greater safety (Gordon *et al.*, 2011).

There are two infectious forms of VACV, the intracellular mature virus (IMV) and the extracellular enveloped virus (EEV), which have different numbers of membranes and distinct surface antigens (Roberts & Smith, 2008). IMV has a single membrane, whereas EEV has a second membrane and promotes spread within an infected host. Despite studies showing that antibodies against EEV are important for protection against disease (Boulter & Appleyard, 1973; Law *et al.*, 2005), immune responses against EEV have been less intensively studied than those against IMV. There are multiple targets for neutralizing antibodies on the IMV surface, including A27 and H3 (Davies *et al.*, 2005; Pütz *et al.*, 2006), but B5 is the only target of EEV-neutralizing antibodies (Bell *et al.*, 2004; Pütz *et al.*, 2006), and is conserved in all strains of variola virus that have been sequenced (Aguado *et al.*, 1992; Massung *et al.*, 1994; Shchelkunov *et al.*, 1994, 1995; Esposito *et al.*, 2006). B5 is also important for virus spread from cell to cell and for virulence (Engelstad *et al.*, 1992; Isaacs *et al.*, 1992; Engelstad & Smith, 1993; Wolffe *et al.*, 1993). The production of only a truncated B5 protein by LC16m8 is therefore relevant to the efficacy of this virus as a vaccine for smallpox, although, in animal models, LC16m8 induces neutralizing antibodies against both IMV and EEV and can protect from a lethal orthopoxvirus challenge (Empig *et al.*, 2006; Meseda *et al.*, 2009). A recent study of the immunogenicity of LC16m8 in man, investigated the seroconversion rate of vaccinees and IMV-neutralizing antibody titres (Saito *et al.*, 2009), but the immunity to individual antigens, including those specific to EEV, and the ability to neutralize EEV remain unknown.

Here, the antibody responses to four VACV antigens were measured by ELISA and the IMV- and EEV-neutralizing titres were determined by plaque reduction assay, as described previously (Pütz *et al.*, 2005, 2006). The antigens selected were the IMV-surface proteins (A27 and H3) and the EEV-surface proteins (B5 and A56), which were produced and purified from bacterial (A27 and H3) or mammalian (B5 and A56) expression systems (Pütz *et al.*, 2006; Midgley *et al.*, 2008). The total anti-VACV antibody titre was also measured by ELISA against the VACV strain Western Reserve (WR)-infected cell lysates (Pütz *et al.*, 2006; Midgley *et al.*, 2008). Serum samples (pre-vaccination and 1 and 5 months after vaccination) were obtained from 42 primary vaccinees and 43 persons vaccinated previously, most likely with the VACV strain Lister (revaccinees), as described previously (Saito *et al.*, 2009). The pre-vaccination sera from primary vaccinees were used to calculate cut-off titres defining seropositivity, defined as three times the geometric mean titre (GMT) of the pre-vaccinated sera. The cut-off titre for each antigen was defined as the maximum dilution of serum that gave a positive-antibody response; these were: B5, 1 : 28; A56, 1 : 63; A27, 1 : 145; H3, 1 : 254; VACV, 1 : 82 and are shown by the dashed line in Fig. 1. The vast majority of pre-vaccination sera were below the cut-offs, with the following specificities: B5, 81 %; A56, 98 %; A27, 83 %; H3, 83 %; VACV, 90 %. Any values below this cut-off were deemed seronegative and given an arbitrary value of one-half of that titre to allow calculations of GMT and to determine effective seroconversion or boosting.

**Fig. 1.**
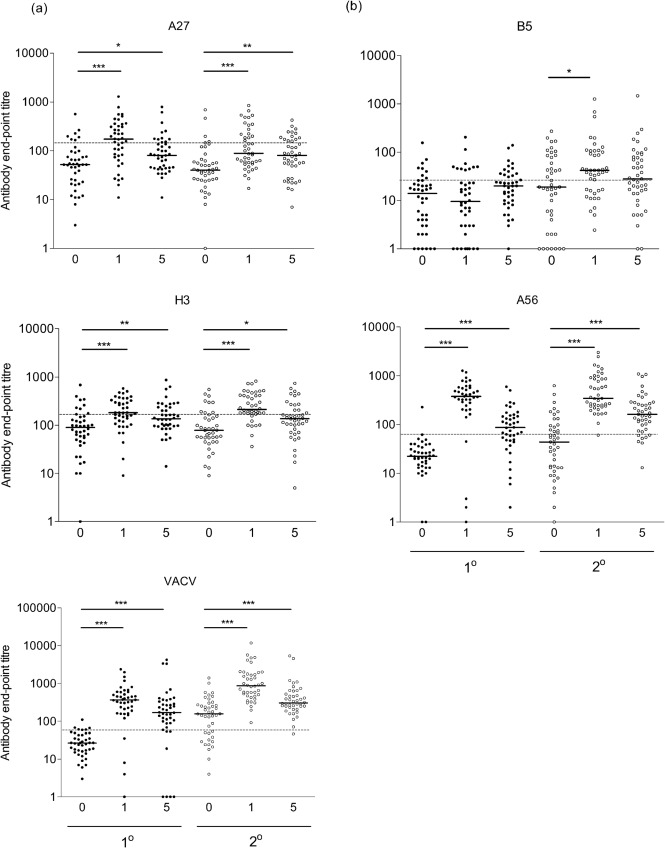
Antibody responses in humans after LC16m8 vaccination. Antibody end-point titres against (a) IMV and (b) EEV proteins were detected pre-vaccination (0) and 1 and 5 months post-vaccination for primary vaccinees (filled circles) and revaccinees (open circles) by ELISA as described in Pütz *et al.* (2006). IgG end-point titres were defined as the reciprocal serum dilution giving twice the average optical density obtained from BSA. A control serum from an individual vaccinated multiple times was used to normalize end-point titres between plates and assays (titres: B5, 1 : 809; A56, 1 : 1213; A27, 1 : 563; H3, 1 : 394; VACV, 1 : 5785). Median values of whole population (black bars), cut-off titres for seropositivity (dashed line) and significant differences between groups, as determined by Mann–Whitney test (*P<0.05, **P<0.005, ***P<0.0001) are shown.

Following vaccination the greatest increase in antibody titre was for antibodies against A56, and VACV-infected cells in which there was a statistically significant increase in mean antibody titre from the pre-vaccination serum to the 1 and 5 months post-vaccination sera in both primary vaccinees and revaccinees (Fig. 1). There were also significant increases in mean titre for antibodies against A27 and H3, although increases were lower than for A56- and VACV-infected cells. In contrast, no antibody response was detected against B5 in primary vaccinees (*P* = 0.1). However, there was a boosting of B5 responses in revaccinees from pre-vaccination to 1 month post-vaccination (*P* = 0.02). GMTs for each antigen also increased following vaccination of primary vaccinees, with the exception of B5 where no increase was seen (Table 1). An increase in GMTs was seen for all antigens in revaccinees, including B5.

**Table 1.** IgG GMTs GMTs and 95 % confidence intervals are given for each VACV antigen and the total VACV antigen in infected cells in ELISA before and after vaccination for primary vaccinees and revaccinees. The fold increase in GMT from pre-vaccination to 1 month post-vaccination is also given.

Antigen	Primary vaccinees				Revaccinees			
	Pre	1 month	5 months	Fold increase	Pre	1 month	5 months	Fold increase
B5	9 (1–17)	9 (0–20)	18 (9–26)	1.0	16 (0–34)	43 (0–109)	30 (0–97)	2.7
A56	21 (11–31)	288 (189–386)	75 (38–111)	13.7	36 (1–72)	436 (248–624)	166 (89–242)	12.1
A27	48 (18–78)	150 (76–223)	86 (41–132)	3.1	42 (6–79)	115 (57–173)	76 (48–104)	2.7
H3	75 (36–114)	173 (130–215)	145 (95–195)	2.3	82 (45–119)	240 (182–297)	127 (78–176)	2.9
VACV	24 (18–31)	267 (120–414)	130 (0–407)	11.1	122 (40–204)	942 (309–1575)	356 (47–665)	7.7

Rates of seroconversion in primary vaccinees and boosting in revaccinees, defined as a fourfold increase in end-point titre from the pre- to post-vaccination sera, varied for each antigen. In primary vaccinees, the IMV antigens A27 and H3 and total VACV had seroconversion rates of 19.0, 2.4 and 76.2 %, respectively. For the EEV antigens, only 2.4 % of primary vaccinees seroconverted to B5, compared with 85.7 % for A56. For revaccinees, the antibody response against IMV antigens A27 and H3 and total VACV were boosted in 16.7, 9.5 and 69.0 % of vaccinees, respectively. For EEV antigens B5 and A56, an effective booster response was seen in 28.6 and 76.2 % of revaccinees, respectively. The observation that B5 responses are boosted in revaccinees, despite little or no antibody response in primary vaccines, is interesting and is likely attributable to the production of a short fragment of the B5 protein up to aa 91 (Meseda *et al.*, 2009).

Some sera were also tested for their ability to neutralize IMV and EEV from the VACV strain WR by plaque-reduction neutralization assay (Pütz *et al.*, 2006). A statistically significant increase in neutralizing antibody titres against IMV was seen from the pre-vaccination sera to the 1 month post-vaccination sera for both primary vaccinees and revaccinees (*P* = 0.0002 and *P* = 0.0043, respectively; Fig. 2). There was also a significant increase in IMV-neutralizing antibody titres from the pre-vaccination sera to the 5 months post-vaccination sera for primary vaccinees (*P* = 0.018). However, there was not a significant increase for revaccinees for this time point (*P* = 0.13). Neutralizing antibody titres correlated well with end-point titres against IMV antigens. In contrast, sera from primary vaccinees did not neutralize EEV, except for one sample that showed a very weak response (Fig. 2b). This correlated with the weak or no anti-B5 responses and was in contrast to the high titres of anti-A56 antibodies. It was also consistent with the prior observation that B5 is the only target for EEV-neutralizing antibody (Bell *et al.*, 2004; Pütz *et al.*, 2006). Several sera from revaccinees neutralized EEV before and after vaccination with LC16m8 (Fig. 2b). In individuals for whom pre-, 1 and 5 months post-vaccination sera were tested, four of eight showed effective boosting of EEV-neutralizing antibodies following LC16m8 vaccination (Fig. 2c), perhaps due to the presence of a truncated B5 protein. It is worth noting that non-neutralizing antibodies against EEV, such as those against A56, may activate the complement system (Benhnia *et al.*, 2009).

**Fig. 2.**
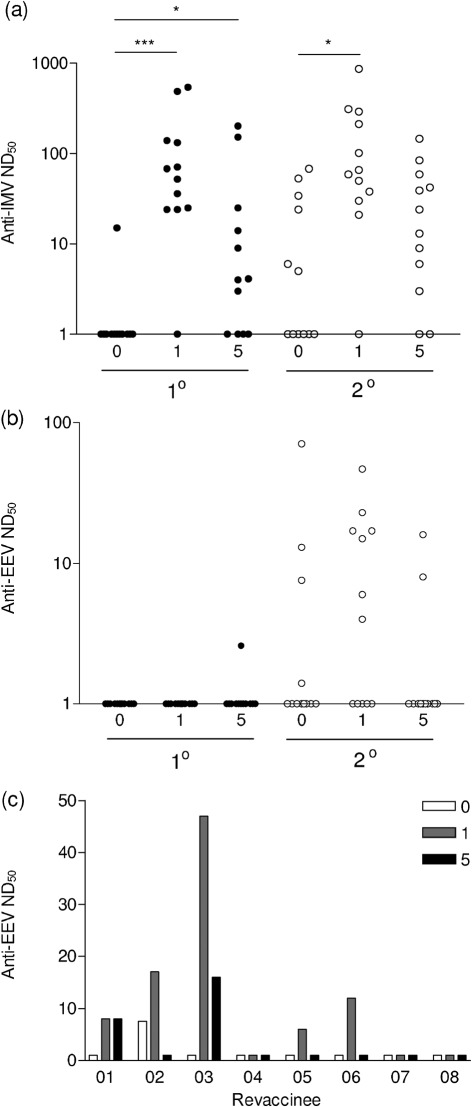
Neutralizing antibody responses were detected by plaque-reduction neutralization against (a) IMV and (b) EEV for pre-vaccination (0) and 1 and 5 months post-vaccination sera from primary vaccinees (filled circles) and revaccinees (open circles) as described in Pütz *et al.* (2006). (c) Shows the response against EEV from eight individual vaccinees. IMV from the VACV strain WR was purified from sucrose density gradients, whereas EEV was harvested from cell supernatant following 24 h post-infection and then incubated with anti-IMV antibody (raised against L1 and A27). ND_50_ values were defined as the reciprocal of the dilution of serum giving a 50 % reduction in plaque number. Significant differences between groups were determined by Mann–Whitney test and shown (*P<0.05, ***P<0.0001).

A comparison of antibody titres in pre-vaccination sera between primary vaccinees and revaccinees showed residual antibody from prior immunization. Differences in the median end-point titres were seen for A56 (*P* = 0.029), VACV (*P*<0.0001) and B5, although the latter was not statistically significant (*P* = 0.085). Pre-existing antibodies were not detected for A27 (*P* = 0.40) or H3 (*P* = 0.86) although these sera did neutralize IMV and EEV, as seen by high titres of neutralizing antibodies in pre-vaccination sera from some revaccinees (Fig. 2). The smallpox eradication campaign in Japan ceased in 1976, showing that these immune responses are still active at least 35 years after vaccination.

Antibody responses to LC16m8 were qualitatively and quantitatively different from those seen for Lister, the VACV strain used most widely in the smallpox eradication campaign. In primary Lister vaccinees, the fold-increases in GMT against antigens B5, A27, H3 and VACV were 13.7, 10.0, 1.8 and 17.1, respectively (Pütz *et al.*, 2006); all but H3 were higher than the responses to LC16m8 (Table 1). LC16m8 also induced lower responses than those following primary Dryvax inoculation [fold increases of B5, 18.8; A27, 17.2; H3, 5.7; VACV, 18.1 (Midgley *et al.*, 2008)]. However, LC16m8 performed favourably compared to the attenuated vaccine NYVAC (B5, 2.2; A27, 1.0; H3, 1.8; VACV, 1.9), apart from in its anti-B5, and therefore anti-EEV, responses (Midgley *et al.*, 2008). In comparison, the human antibody responses to MVA are lower than those to Dryvax in primary vaccinees, but similar in revaccinees (Davies *et al.*, 2007; Parrino *et al.*, 2007). Overall, LC16m8 induces quantitatively lower antibody responses in primary vaccinees than Lister or Dryvax, but stronger responses than NYVAC. LC16m8 also induces qualitatively different responses to each of these vaccines, as shown by the absence of B5- or EEV-neutralizing antibodies in primary vaccinees. It is important to note that LC16m8 has an excellent safety record with less complications and contraindications than either Lister or Dryvax.

In summary, an analysis of the serological responses induced by VACV LC16m8 in primary vaccinees showed that IMV-neutralizing antibodies were induced and there was a good response to the A27 IMV-surface proteins and total VACV antigen from infected cells. In primary vaccinees, LC16m8 failed to induce EEV-neutralizing antibody and consistent with this antibodies to EEV protein B5 were not produced; however, a boosting response against B5 protein was observed in revaccinees. Since B5 is the only EEV antigen that is the target for EEV-neutralizing antibody, and since it is conserved in all strains of variola virus, these data suggest that immunity induced by LC16m8 might be less potent than that deriving from the VACV strain Lister. Nevertheless, several studies showed that, similar to Lister, LC16m8 can protect animals against disease caused by some orthopoxviruses (Empig *et al.*, 2006; Meseda *et al.*, 2009). In addition, it should be noted that the exact correlates of protection against smallpox remain uncertain, and that there is evidence for involvement of both antibodies and cellular immunity in protection against orthopoxviruses, for review see Moss (2011). The potential disadvantage of reduced immunogenicity of LC16m8 should be considered together with the advantage of increased safety of this vaccine.
